# Surgeon-Perceived Difficulty in Robot-Assisted Radical Prostatectomy Using the Hinotori System: A Multicenter Survey Study

**DOI:** 10.7759/cureus.90487

**Published:** 2025-08-19

**Authors:** Takuto Hara, Kotaro Suzuki, Hideto Ueki, Naoto Wakita, Yasuyoshi Okamura, Yukari Bando, Tomoaki Terakawa, Koji Chiba, Jun Teishima, Hideaki Miyake

**Affiliations:** 1 Division of Urology, Department of Surgery, Kobe University Graduate School of Medicine, Kobe, JPN

**Keywords:** hinotori surgical robot system, multicenter survey study, robot-assisted radical prostatectomy, robotic surgery training, surgeon-perceived difficulty

## Abstract

Objectives

The hinotori™ Surgical Robot System (Medicaroid Corporation, Kobe, Japan) offers advanced capabilities for performing complex urological surgeries. However, despite its growing adoption, limited data are available on surgeons' subjective impressions of its usability and the process of skill acquisition. This study aimed to assess surgeons' perceptions of the difficulty of key surgical steps during robot-assisted radical prostatectomy (RARP) using the hinotori system.

Methods

We included facilities that perform 20 or more RARP procedures annually using the hinotori system. In November 2024, an anonymous online survey was conducted among urologists at these facilities to collect information on their overall RARP experience and specific experience with the hinotori system. Surgeons used a five-point Likert scale to rate the difficulty of seven surgical steps. Although this approach is unique, several steps, such as bladder neck dissection, nerve sparing, and vesicourethral anastomosis, are also considered technically demanding in studies with other robotic systems. Statistical analyses were performed to assess differences based on experience levels.

Results

We received 58 survey responses, representing a cumulative experience of over 800 RARP cases performed using the hinotori system. Bladder takedown was perceived as the least difficult step, whereas bladder neck dissection, nerve sparing, vesicourethral anastomosis, and pelvic lymph node dissection were rated as more challenging. Stratification by experience showed that surgeons with 40 or more hinotori RARP cases perceived bladder takedown (p = 0.033), nerve sparing (p = 0.024), and vesicourethral anastomosis (p = 0.001) as being easier.

Conclusion

Surgeons' prior experience with other robotic systems influenced the perceived difficulty of procedures using the hinotori system, particularly for complex steps such as nerve-sparing and vesicourethral anastomosis.

## Introduction

Robot-assisted surgeries have revolutionized various medical fields, including urology, general surgery, gynecology, and thoracic surgery, by providing minimally invasive techniques and improving functional preservation. In urology, procedures such as radical prostatectomy and partial nephrectomy have particularly benefited from robot assistance, contributing to the global adoption of robotic surgical systems. This technological advancement aligns with the increasing demand for high-precision surgeries that optimize patient outcomes while minimizing recovery times.

The global surgical robot systems market was valued at USD 11.48 billion in 2024 and is projected to grow at a compound annual growth rate of 12.4% from 2025 to 2030 [1]. This growth reflects the rapid global adoption of robotic-assisted surgeries, driven by innovations in surgical technology and expanding clinical indications. To meet this growing demand, companies in various countries are actively developing and commercializing innovative surgical support robots. Several advanced robotic systems are now available for clinical use in urological surgeries in Japan. These include the da Vinci Surgical System (Si, X, Xi), the da Vinci SP Surgical System, the Hugo RAS System, and the Saroa Surgical System. The hinotori™ Surgical Robot System (Medicaroid Corporation, Kobe, Japan), developed in Japan, holds a unique position with features tailored to the needs of domestic surgical practice.

The hinotori™ Surgical Robot System was launched in Japan in 2020. The system stands out due to its unique features, including compact robotic arms with eight axes of motion for enhanced flexibility and precision. It employs software-based calibration for trocar positioning, eliminating the need for manual docking and reducing the risk of collisions. The surgical cockpit is equipped with a high-definition 3D viewer, offering a wide visual field to support surgical precision and minimize surgeon fatigue. These attributes make hinotori particularly well-suited for complex urological cancer surgeries [2].

As of September 2024, 64 hinotori systems were operational in Japan, and the system has also been introduced in other countries such as Malaysia and Singapore [3]. There is limited data on surgeons' subjective impressions, such as ease of use and skill acquisition, specific to the hinotori system. Understanding these perceptions is essential for optimizing the system’s usability, improving training programs, and ultimately enhancing surgical outcomes.

To our knowledge, this is the first multicenter survey to evaluate step-specific surgeon-perceived difficulty in robot-assisted radical prostatectomy (RARP) using the hinotori system. To address this gap, we conducted an anonymous online survey targeting urologists performing more than 20 RARP procedures annually at facilities equipped with the hinotori system. The survey assessed the perceived difficulty of each surgical step during RARP, aiming to further elucidate patterns in difficulty perception and their relationship to surgeon experience, thereby providing insights into the usability of the hinotori system. This study provides novel insights into the surgeons’ experience and contributes to the development of evidence-based training protocols for this robotic platform.

## Materials and methods

In 2023, we identified facilities that performed 20 or more RARP procedures annually using the hinotori system. In November 2024, we conducted an anonymous online survey targeting urologists at these facilities. Representatives from each facility were asked to distribute a link to the survey to their surgeons. Participation was voluntary, and multiple responses from the same institution were permitted.

The survey collected anonymous information on years of experience in urology, total RARP experience across all surgical systems, and RARP experience specifically using the hinotori system. Based on a transperitoneal non-Retzius-sparing approach, which is the most commonly used technique for RARP with the hinotori system in Japan and was standardized across participating sites, participants rated the perceived difficulty of key surgical steps: bladder takedown, bladder neck dissection, prostate-rectum separation, nerve sparing, apical dissection, vesicourethral anastomosis, and pelvic lymph node dissection. Difficulty levels were evaluated on a five-point Likert scale, ranging from 1 (not difficult at all) to 5 (extremely difficult). The survey form is presented in Table 1 to facilitate readers’ understanding of the methodology. Surgeons were categorized into two groups according to their experience with the hinotori system: fewer than 39 cases and 40 or more cases. The threshold of 40 cases was chosen in reference to the proctorship certification criteria established by the Japanese Society of Endourology and Robotics [4]. In further subgroup analyses, both total RARP experience and hinotori-specific experience were considered to distinguish overall proficiency in robotic-assisted prostatectomy from familiarity with the hinotori system, as no previous studies have evaluated this distinction.

**Table 1:** Survey questionnaire used in the study.

Survey questionnaire	
How many years have you been practicing as a urologist?	
・0-9 years	
・10-19 years	
・20-29 years	
・30- years	
How much experience do you have with robotic-assisted total prostatectomy (RARP)?	
・1-9	
・10-39	
・40-99	
・100-	
How many RARPs have you performed using the hinotori surgical system?	
・1-9	
・10-39	
・40-99	
・100-	
Please answer with the transperitoneal non-Retzius-sparing approach in mind. How do you evaluate the difficulty level of each part of the RARP when using the hinotori system?	
1 = Do not find it difficult at all. 5 = Extremely difficult	
Bladder take down	1, 2, 3, 4, 5, No answer
Bladder neck dissection	1, 2, 3, 4, 5, No answer
Dissection between the rectum and the prostate	1, 2, 3, 4, 5, No answer
Apical dissection	1, 2, 3, 4, 5, No answer
Urethral vesical anastomosis	1, 2, 3, 4, 5, No answer
Nerve sparing	1, 2, 3, 4, 5, No answer
Pelvic lymphadenectomy	1, 2, 3, 4, 5, No answer

As this study was based on an anonymous survey, the need for ethical review was confirmed with the Kobe University Institutional Review Board and was determined to be waived. Ethical considerations were carefully observed by ensuring anonymity and confidentiality throughout the data collection and analysis processes.

Statistical analyses were performed using EZR version 1.68 (Saitama Medical Center, Jichi Medical University, Saitama, Japan) [5], which is a graphical user interface for R version 4.3.1 (R Foundation for Statistical Computing, Vienna, Austria). The Mann-Whitney U test was applied for non-parametric comparisons between two groups, and the Kruskal-Wallis test was used for comparisons among three groups. Pairwise comparisons among surgical steps were adjusted using the Bonferroni correction. Comparisons between experience groups for each surgical step are shown as unadjusted p-values.

## Results

Between January and December 2023, 23 facilities conducted 20 or more RARP procedures, accounting for about 800 cases.

We received 61 survey responses, of which 58 were included in the analysis. Three cases were excluded because they lacked responses for all surgical step evaluations. The urology experience of the respondents was categorized as follows: less than nine years (one respondent, 1.7%), 10-19 years (16 respondents, 27.6%), 20-29 years (33 respondents, 56.9%), and over 30 years (eight respondents, 13.8%). The number of RARP cases using the hinotori system was classified as fewer than 39 cases (37 respondents) and 40 or more cases (21 respondents). Among those with fewer than 39 hinotori cases, nine had fewer than 39 total RARP cases, 18 had 40-99 cases, and 10 had over 100 cases. Of the 21 respondents with more than 40 hinotori cases, 17 (76.4%) had performed over 100 RARP procedures. The Supplemental Table shows detailed survey results.

**Figure 1:**
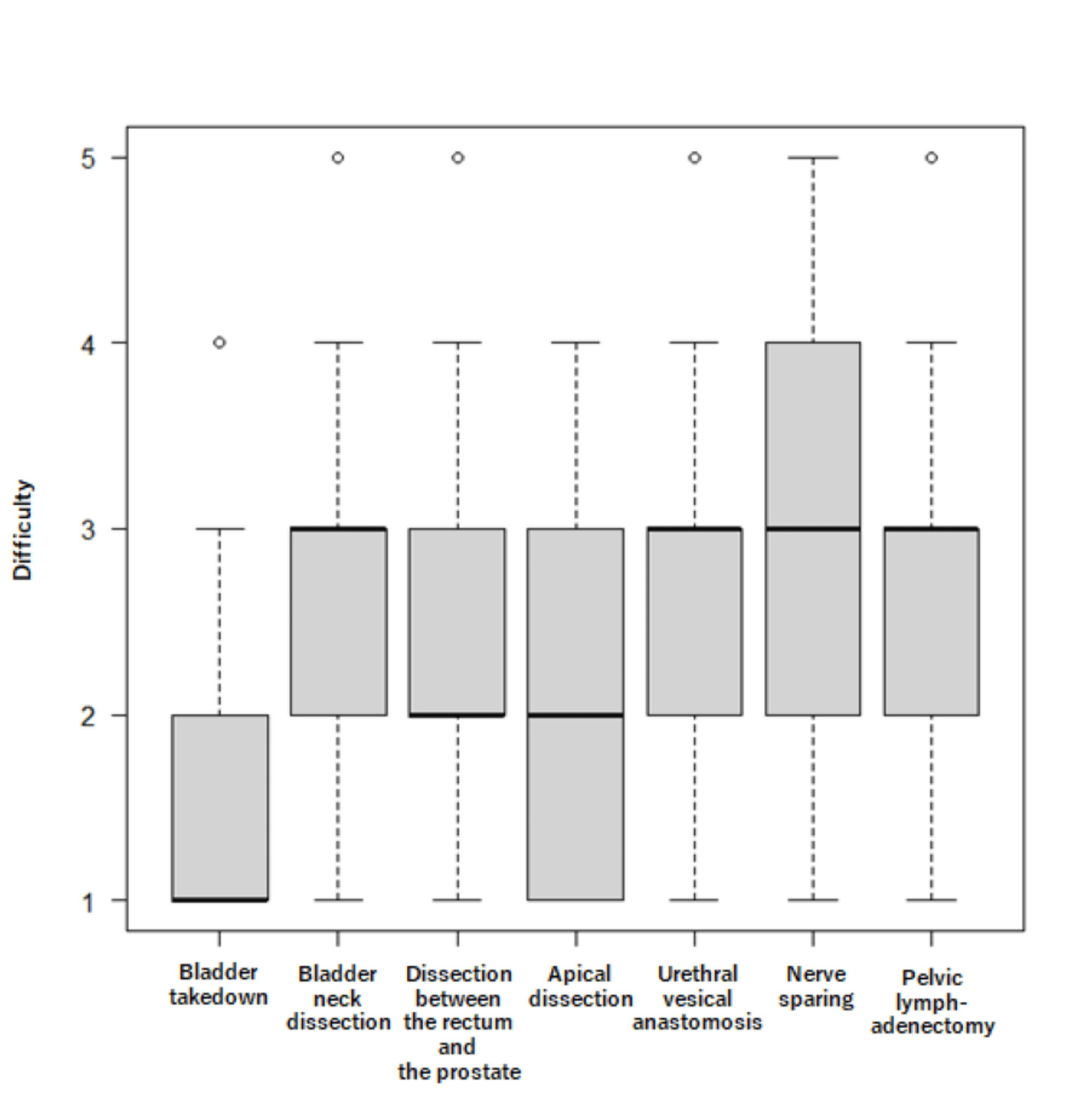
Difficulty levels of surgical steps in robot-assisted radical prostatectomy (RARP) using the hinotori surgical robot system. Boxplots illustrate the perceived difficulty levels for each surgical step based on a five-point Likert scale (1: not difficult at all; 5: extremely difficult). Median values are indicated by bold horizontal lines, and interquartile ranges (IQR) are shown as shaded boxes. Whiskers represent the range, excluding outliers, which are displayed as open circles.

Figure 1 and Table 2 summarize the difficulty levels reported for each surgical step. Bladder takedown was rated as significantly easier than each of the other surgical steps in pairwise comparisons. Prostate-rectum separation and apical dissection were perceived as easier than bladder neck dissection and nerve sparing. No significant differences were observed for other steps. Bladder neck dissection, nerve sparing, vesicourethral anastomosis, and pelvic lymph node dissection were generally perceived as more challenging (Table 3).

**Table 2:** Difficulty levels reported for each surgical step.

	1 - Not difficult at all	2	3	4	5 - Extremely difficult	No answer
Bladder takedown (%)	39 (67.2)	13 (22.4)	3 (5.2)	3 (5.2)	0	0
Bladder neck dissection (%)	7 (12.1)	14 (24.1)	23 (39.7)	8 (13.8)	6 (10.3)	0
Prostate-rectum separation (%)	12 (20.7)	26 (44.8)	17 (29.3)	2 (3.4)	1 (1.7)	0
Nerve sparing (%)	5 (8.6)	19 (32.8)	14 (24.1)	15 (39.5)	2 (3.4)	3 (5.2)
Apical dissection (%)	15 (25.9)	22 (37.9)	17 (29.3)	4 (6.9)	0	0
Vesicourethral anastomosis (%)	12 (20.7)	26 (44.8)	17 (29.3)	2 (3.4)	1 (1.7)	0
Pelvic lymph node dissection (%)	7 (12.1)	13 (22.4)	21 (36.2)	6 (10.3)	6 (10.3)	5 (8.6)

**Table 3:** Pairwise comparisons of perceived surgical step difficulty using the hinotori system. * Significant (p-values adjusted using the Bonferroni correction).

	Bladder take down	Bladder neck dissection	Dissection between the rectum and the prostate	Apical dissection	Urethral vesical anastomosis	Nerve sparing	Pelvic lymphadenectomy
Bladder take down	-	-	-	-	-	-	-
Bladder neck dissection	<0.001*	-	-	-	-	-	-
Dissection between the rectum and the prostate	<0.001*	0.020*	-	-	-	-	-
Apical dissection	<0.001*	0.019*	1.000	-	-	-	-
Urethral vesical anastomosis	<0.001*	1.000	0.527	0.454	-	-	-
Nerve sparing	<0.001*	1.000	0.040*	0.033*	1.000	-	-
Pelvic lymphadenectomy	<0.001*	1.000	0.054	0.051	1.000	1.000	-

**Figure 2:**
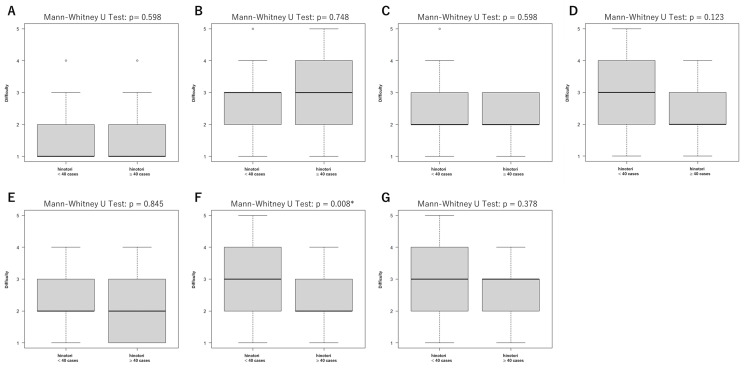
Boxplots of perceived difficulty levels for each surgical step during robot-assisted radical prostatectomy (RARP) using the hinotori Surgical Robot System. Ratings are based on a five-point Likert scale (1: not difficult at all; 5: extremely difficult). Median values are indicated by bold horizontal lines, interquartile ranges (IQR) are shown as shaded boxes, and whiskers represent the range, excluding outliers (displayed as open circles). The analyzed steps include bladder takedown (A), bladder neck dissection (B), prostate-rectum separation (C), nerve sparing (D), apical dissection (E), vesicourethral anastomosis (F), and pelvic lymph node dissection (G). Mann-Whitney U tests are performed to compare the distributions of perceived difficulty between groups with fewer than 40 cases and 40 or more cases for each surgical step. P-values are unadjusted. Bonferroni-adjusted p-values for comparisons among surgical steps are presented separately in Table 3.

As shown in Figure 2, surgeons who performed 40 hinotori cases or more reported significantly less difficulty in nerve sparing (p = 0.008). No significant differences were observed for other steps.

**Figure 3:**
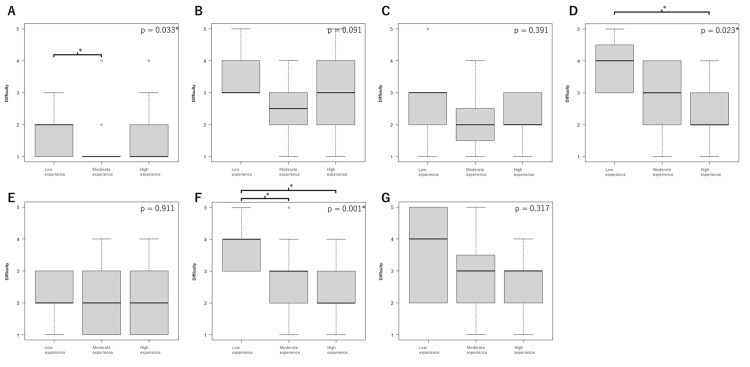
Boxplots of perceived difficulty levels for each surgical step during robot-assisted radical prostatectomy (RARP) using the hinotori Surgical Robot System. Ratings are based on a five-point Likert scale (1: not difficult at all; 5: extremely difficult). Median values are indicated by bold horizontal lines, interquartile ranges (IQR) are shown as shaded boxes, and whiskers represent the range, excluding outliers (displayed as open circles). The analyzed steps include bladder takedown (A), bladder neck dissection (B), prostate-rectum separation (C), nerve sparing (D), apical dissection (E), vesicourethral anastomosis (F), and pelvic lymph node dissection (G). Respondents were divided into three groups based on experience: low experience (fewer than 39 hinotori cases and fewer than 39 total RARP cases, nine respondents), moderate experience (fewer than 39 hinotori cases but 40 or more total RARP cases, 28 respondents), and high experience (40 or more hinotori cases, 21 respondents). Kruskal-Wallis test was performed to compare the distributions of perceived difficulty among the three groups, followed by Bonferroni-corrected pairwise comparisons for post-hoc analysis. P-values are unadjusted. Bonferroni-adjusted p-values for comparisons among surgical steps are presented separately in Table 2.

Respondents were divided into three groups based on their experience levels for further analysis: low experience (fewer than 39 hinotori cases and fewer than 39 total RARP cases, nine respondents), moderate experience (fewer than 39 hinotori cases but 40 or more total RARP cases, 28 respondents), and high experience (40 or more hinotori cases, 21 respondents). Figure 3 shows the results of the surgeon’s perceived difficulties with each step. There were significant differences in bladder takedown among the groups (p = 0.033), with the moderate experience group finding it easier than the low experience group (p = 0.032). There were significant differences for nerve sparing among the groups (p = 0.024), especially between the low and high experience groups (p = 0.025). Moreover, the moderate group reported reduced difficulty in nerve sparing; however, the difference was not significant (p = 0.075). Vesicourethral anastomosis showed significant differences among the groups (p = 0.001), with both moderate (p = 0.036) and high groups (p = 0.001) finding it easier than the low group.

## Discussion

The hinotori™ Surgical Robot System is being increasingly adopted in Japan; however, there has been limited investigation into the development of surgeons' skills when using this system. A previous single-center study examined the initial learning curve of RARP performed with hinotori [6]; however, our study is novel in that it evaluated step-specific perceived intraoperative difficulty through a multicenter survey, rather than focusing solely on learning curves or surgical outcomes at single high-volume centers. Previous comparisons of surgical outcomes [7-9] primarily focused on high-volume centers, such as university hospitals, in Japan. In contrast, our study was a nationwide analysis, including general urologists. Our findings may contribute to the development of future surgical education systems and improvements in robotic systems.

We found that surgeons generally perceive bladder takedown to be less challenging when using the hinotori™ Surgical Robot System for RARP. In contrast, bladder neck dissection, nerve sparing, vesicourethral anastomosis, and pelvic lymph node dissection were consistently rated as more difficult. These findings support previous studies that reported that steps such as bladder neck dissection [10,11], nerve sparing [12], and vesicourethral anastomosis [13] are technically demanding for robotic prostatectomy, regardless of the system employed. However, the current data offer a more detailed perspective on the hinotori system’s usability, highlighting a steep learning curve for complex steps and providing quantitative insights into how perceived difficulty evolves with experience.

Figures 2, 3 show non-parametric methods used to evaluate the perceived difficulty of each step based on robotic surgery experience. Figure 2 focuses on the number of cases performed with the hinotori system, showing that only the nerve-sparing step demonstrated a significant reduction in perceived difficulty for surgeons with more than 40 cases. Among these surgeons (n = 21), 17 (80.9%) had experience with over 100 cases of RARP using other robotic systems (likely the da Vinci system, given its prevalence in Japan). Conversely, among surgeons with fewer than 40 cases of RARP using the hinotori system, experience varied widely, ranging from fewer than 10 cases to over 100 cases of RARP using other robotic systems.

Figure 3 compares surgeon groups stratified by both total RARP experience and hinotori-specific experience among those with fewer than 40 hinotori cases. They were further subdivided into three groups based on their experience. The results showed clearer differences depending on experience. Significant differences were observed not only for nerve sparing but also for vesicourethral anastomosis. Notably, dividing nerve sparing into three groups demonstrated stronger p-values compared with a two-group classification. This suggests that experience with other surgical systems positively influenced mastery of a specific system, indicating the existence of shared surgical skills transferable across platforms. Our finding that prior experience with other robotic systems appears to facilitate mastery of the hinotori system supports previous studies [14,15], which reported that proficiency in the da Vinci system facilitated a smooth transition to the Hugo system and suggested a similar trend for the hinotori system. Nonetheless, differences between the hinotori and da Vinci systems in console operation, instrument range of motion, and system ergonomics, such as variations in arm articulation, clutching behavior, and camera control, necessitate focused training and adaptation. Future studies are needed to examine whether structured modular training and proctorship can expedite the learning curve for surgeons experienced with other platforms.

Our study demonstrated that surgical experience significantly influences perceived difficulty in nerve sparing, which is crucial for postoperative erectile function, and in vesicourethral anastomosis, which impacts urinary continence. These results align with previous studies [16-18] that reported that surgical experience has an effect on postoperative erectile function and continence preservation. On the other hand, for bladder neck dissection, another high-difficulty step affecting continence, no significant differences were observed between low- and high-experience groups in terms of mean values and variability. While increased experience may expand the range of approaches to accommodate tumor location and progression [19], this variability in approaches may also pose additional challenges for surgeons. However, as this study was not designed to compare robotic systems, further research is needed to attribute these findings to hinotori system-specific characteristics.

Several limitations of this study should be acknowledged. First, the survey relied on subjective self-assessments rather than objective performance metrics, potentially introducing bias. In particular, we did not collect detailed data on console training hours, the expertise of individual surgeons, or their surgical outcomes and complication rates, which could provide further context in future studies. Additionally, the definitions of each step were left to the interpretation of the respondents, which may have introduced variability in responses. Future surveys could incorporate visual or procedural anchors, such as standardized video clips or detailed step descriptions, to improve consistency and reduce subjectivity. Moreover, this study was not designed as a direct comparison with other robotic platforms, such as the da Vinci system; future head-to-head comparisons could further elucidate platform-specific challenges and transferable skills. Second, although responses were obtained from facilities performing at least 20 RARP cases annually using the hinotori system, the total number of eligible surgeons remains unknown. As the response rate was unclear, there may have been selection bias. However, it should be noted that the survey was conducted within the context of about 3,200 hinotori-assisted RARP cases performed nationwide as of November 2024, and data from more than 800 cases were collected, lending a degree of robustness and representativeness to the findings. Nevertheless, potential selection bias and uncertainty regarding the response rate may limit the generalizability of these results. Third, the cross-sectional design captures perceptions at a single time point, lacking longitudinal follow-up to assess changes as case volumes increase. Lastly, variations in patient characteristics, surgical complexity, and the presence of proctors or additional staff support may have influenced perceptions of difficulty.

## Conclusions

In conclusion, our findings suggest that prior experience with other robotic systems influences the perceived difficulty of procedures performed using the hinotori system. Surgeons with greater experience found complex steps, such as nerve sparing and vesicourethral anastomosis, less challenging, indicating that skills acquired from other platforms may be transferable. Further studies incorporating objective performance metrics and longitudinal assessments are necessary to evaluate learning curves and refine training protocols, including structured simulation and modular training, for optimizing outcomes as the hinotori system becomes more widely adopted.

## Appendices

**Table 4:** Summary of survey responses. RARP: robot-assisted radical prostatectomy.

Year	RARP experience	Hinotori experience	Bladder takedown	Bladder neck dissection	Prostate-rectum separation	Apical dissection	Vesicourethral anastomosis	Nerve-sparing	Pelvic lymph node dissection
10-19	-9	-9	2	3	2	2	3	NA	NA
10-19	-9	-9	2	4	5	3	3	NA	NA
10-19	10-39	-9	1	3	2	1	3	4	3
10-19	10-39	-9	1	3	3	2	5	5	5
10-19	40-99	-9	1	1	1	1	1	1	2
10-19	40-99	-9	1	3	2	2	3	2	2
10-19	40-99	-9	1	2	2	2	4	3	4
10-19	40-99	-9	1	3	2	2	5	4	3
20-29	40-99	-9	1	2	1	1	2	2	2
20-29	40-99	-9	1	3	2	2	3	2	2
20-29	40-99	-9	1	3	3	2	2	4	4
20-29	40-99	-9	1	3	3	2	3	4	3
20-29	40-99	-9	1	3	2	2	5	4	3
20-29	100-	-9	1	4	3	1	1	3	2
1-9	10-39	10-39	3	3	3	3	4	4	NA
10-19	10-39	10-39	2	3	1	2	4	3	2
10-19	10-39	10-39	2	3	1	2	4	3	2
10-19	10-39	10-39	1	4	3	2	5	5	5
20-29	10-39	10-39	3	5	3	3	3	3	5
20-29	10-39	10-39	NA	NA	NA	NA	NA	NA	NA
20-29	10-39	10-39	NA	NA	NA	NA	NA	NA	NA
10-19	40-99	10-39	1	2	1	1	2	3	1
10-19	40-99	10-39	2	4	2	4	3	2	3
20-29	40-99	10-39	1	2	1	1	1	1	1
20-29	40-99	10-39	1	2	2	3	1	2	3
20-29	40-99	10-39	1	2	2	3	1	2	3
20-29	40-99	10-39	1	2	2	2	3	2	1
20-29	40-99	10-39	2	2	2	3	3	3	2
20-29	40-99	10-39	2	2	2	3	3	NA	2
20-29	40-99	10-39	1	4	3	2	3	3	5
10-19	100-	10-39	4	3	2	2	3	4	5
20-29	100-	10-39	1	1	2	1	1	2	1
20-29	100-	10-39	1	2	3	3	3	4	4
20-29	100-	10-39	1	3	1	3	4	2	4
20-29	100-	10-39	4	3	2	2	2	4	5
20-29	100-	10-39	1	4	4	4	3	4	3
20-29	100-	10-39	1	4	4	4	4	4	3
30-	100-	10-39	1	2	1	1	3	2	3
30-	100-	10-39	1	2	1	1	3	2	3
10-19	40-99	40-99	2	5	2	4	2	4	NA
10-19	40-99	40-99	2	5	3	3	2	4	3
20-29	40-99	40-99	1	5	3	3	4	4	4
20-29	40-99	40-99	1	5	3	3	4	4	4
20-29	40-99	40-99	NA	NA	NA	NA	NA	NA	NA
20-29	100-	40-99	1	1	1	1	1	1	1
20-29	100-	40-99	1	1	3	1	1	2	2
20-29	100-	40-99	1	1	3	1	1	2	2
20-29	100-	40-99	3	3	2	3	2	3	3
20-29	100-	40-99	4	3	2	3	2	3	3
30-	100-	40-99	1	3	2	1	2	2	1
30-	100-	40-99	2	3	2	2	2	2	3
30-	100-	40-99	2	3	2	2	3	2	3
20-29	100-	100-	1	2	2	2	2	3	3
20-29	100-	100-	1	2	2	2	2	3	3
20-29	100-	100-	2	3	2	2	2	2	2
20-29	100-	100-	1	3	3	3	3	3	3
20-29	100-	100-	1	4	3	3	3	2	3
20-29	100-	100-	1	5	3	3	3	3	3
30-	100-	100-	1	1	1	1	1	1	NA
30-	100-	100-	1	1	1	1	1	1	1
30-	100-	100-	2	3	2	2	2	2	2
